# Sleep Quality and Falls Risk in the ELSA Study: A Cross‐Sectional and Prospective Cohort Study

**DOI:** 10.1002/brb3.71719

**Published:** 2026-08-26

**Authors:** Yiwei Li, Yifa Rong, Kai Jiang, Bowen Lu, Xuezhen Liang, Jiacheng Li, Gang Li

**Affiliations:** ^1^ The First Clinical Medical School Shandong University of Traditional Chinese Medicine Shandong China

**Keywords:** depressive symptoms, ELSA, fall, mediation analysis, sleep quality

## Abstract

**Background:**

With aging populations, falls represent a significant public health challenge. Previous studies suggest that poor sleep quality may increase fall risk, but evidence among adults aged 50 years or older in the UK remains limited. The present study sought to investigate the associations between sleep quality, falls, and new‐onset falls, along with potential underlying mechanisms.

**Methods:**

Data from the English Longitudinal Study of Ageing were analyzed. Cross‐sectional associations between sleep quality and falls were assessed at baseline, and prospective analyses evaluated new‐onset falls over a 4‐year follow‐up period between Wave 4 and Wave 8. Sleep quality was measured using the Jenkins Sleep Scale. Logistic regression models and restricted cubic spline (RCS) analyses were used to evaluate associations and dose–response relationships, respectively. Machine learning approaches were applied to identify risk factors for falls, and mediation analyses explored potential underlying pathways.

**Results:**

In the cross‐sectional analysis, 9,734 participants were included (mean age, 68.11 years; 54.34% were female), of whom 2,446 reported falls. After adjustment for covariates, poorer sleep quality was independently associated with greater odds of falls (OR = 1.07, 95% CI: 1.05–1.09, *p* < 0.0001). In the prospective analysis, 3,224 participants were included (mean age, 68.32 years; 53.16% were female), of whom 1,452 reported new‐onset falls. Findings were similar to those observed in the cross‐sectional analysis (OR = 1.04, 95% CI: 1.01–1.07, *p* < 0.05). RCS analyses revealed an increase in fall risk as sleep quality worsened (*p*‐overall < 0.05). LASSO regression and the Boruta algorithm identified depressive symptoms as key variables across different study designs. Mediation analyses indicated that depressive symptoms mediated 28.24% and 28.20% of the associations of sleep quality with falls and new‐onset falls, respectively.

**Conclusions:**

Poor sleep quality is significantly associated with a greater risk of falls among adults aged 50 or older. Depressive symptoms may partially mediate this association. Interventions addressing both sleep quality and depressive symptoms may help reduce falls in this population.

## Graphical abstract

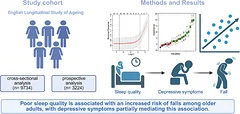

**Abbreviations**
CBT‐I: Cognitive behavioral therapy for insomnia
CES‐D: Centre for Epidemiological Studies Depression Scale
CIs: confidence intervals
ELSA: English Longitudinal Study of Ageing
HPA: hypothalamic‐pituitary‐adrenal
ISI: Insomnia Severity Index
JSS: Jenkins Sleep Scale
LASSO: least absolute shrinkage and selection operator
NS‐SEC: National Statistics Socio‐Economic Classification
ORs: Odds ratios
PSQI: Pittsburgh Sleep Quality Index
RCS: Restricted cubic spline
SD: standard deviation

## 1 Introduction

A fall refers to an event in which a person unintentionally comes to rest on the ground or another lower surface, either from the same level or from a height (Montero‐Odasso et al. 2022). Evidence indicates that fall incidence among individuals aged 65 years or older has increased by 182% since 1990, with the associated burden projected to rise further globally (Chen et al. 2025). Data from the Ardakan cohort showed that 19.9% of middle‐aged and older adults experienced falls, while 10.1% reported two or more fall events (Delbari et al. 2024). Another worldwide analysis reported an estimated pooled prevalence of falls of about 26.5% in older populations (Salari et al. 2022). Falls commonly lead to fractures, substantially impairing quality of life and elevating the risk of death (Hamilton et al. 2024). Within the United States, falls are the leading cause of injury and injury‐related mortality in people aged 65 years or older, placing considerable burdens on affected individuals, their families, and wider society (Kakara et al. 2023). Therefore, falls represent both an important public health issue and a major challenge for aging societies.

Sleep quality is a multidimensional construct that encompasses both subjective perceptions and objective indicators. It is commonly assessed using self‐report instruments, including the Pittsburgh Sleep Quality Index (PSQI), Jenkins Sleep Scale (JSS), and Insomnia Severity Index (ISI). A study based on the Hertfordshire Cohort Study reported that approximately 37% of men and 43% of women experienced sleep disturbances (Bevilacqua et al. 2020). Self‐perceived sleep problems are frequently reported among middle‐aged and older individuals and are associated with unfavorable health outcomes, including cardiovascular disease and diabetes (Lao et al. 2018; LeBlanc et al. 2018; Gadie et al. 2017). In sleep‐related research, sleep quality generally refers to an individual's assessment of their overall sleep status and sleep experience, encompassing multiple dimensions such as sleep satisfaction and sleep characteristics (Krystal and Edinger 2008). Sleep disturbance is commonly used to describe disruptions in sleep or abnormal alterations in sleep patterns (Lee et al. 2009). Insomnia specifically refers to recurrent sleep‐related symptoms, including difficulty initiating sleep, difficulty maintaining sleep, or early morning awakening (Riemann et al. 2015). Sleep disorders represent a broader clinical classification that includes a range of conditions characterized by abnormalities in sleep or sleep–wake regulation, such as sleep‐related breathing disorders, circadian rhythm sleep–wake disorders, and other sleep‐related conditions (Sateia 2014). This study assessed sleep quality with the JSS, rather than treating it as physician‐diagnosed insomnia or another clinically defined sleep disorder. The JSS primarily captures key sleep problems, including trouble initiating sleep and repeated awakenings during the night, and has been recognized as a reliable tool for epidemiological studies of aging populations (Juhola et al. 2021).

The link between sleep quality and fall risk has received increasing research attention in recent years. Among a cohort of 1,071 older adults, 28.9% reported poor sleep quality, which was positively associated with later fall risk in longitudinal analyses (Takada et al. 2018). Similar findings were observed among older adults in Vietnam (Diep et al. 2024). Notably, poor sleep is associated with the occurrence and severity of depressive symptoms. Neurobiological evidence suggests that sleep disturbances may disrupt brain networks involved in cognitive and emotional regulation through mechanisms involving impaired prefrontal cortical function and altered hippocampal‐dependent processes. These neural alterations may compromise executive attention, memory function, and emotional regulation, thereby increasing vulnerability to depressive symptoms. Depressive symptoms may further increase fall risk through cognitive decline, impaired attention, and reduced motor coordination (Yoo et al. 2007; van der Werf et al. 2009). Therefore, sleep quality, depressive symptoms, and falls may be interconnected through complex pathways; specifically, poor sleep may indirectly contribute to falls through its potential influence on depressive symptoms. Although the relationships among sleep quality, falls, and depressive symptoms have attracted considerable attention, few studies have combined machine learning algorithms with mediation analysis to investigate whether depressive symptoms act as an intermediate pathway in these relationships. Moreover, evidence integrating both cross‐sectional and prospective analyses remains limited. Using ELSA data, we examined the associations between sleep quality and fall risk, including new‐onset falls, in a large nationally representative sample of adults aged 50 years or older in England.

We hypothesized that poorer sleep quality would be associated with an increased risk of falls and that key variables identified through machine learning approaches, such as depressive symptoms, might serve as potential mediators of this association. Mediation analyses were subsequently conducted to evaluate these potential mediating pathways. By clarifying these potential pathways, the study may improve understanding of how poor sleep contributes to fall‐related outcomes and inform targeted strategies to reduce fall risk among adults aged 50 years or older.

## 2 Methods

### 2.1 Study Population

Data were drawn from ELSA, a nationally representative UK cohort comprising adults aged 50 years or older. The ELSA dataset was accessed through the UK Data Service, and data management and statistical analyses were conducted using R software (version 4.4.3). Ethical approval for ELSA was granted by the London Multicentre Research Ethics Committee (Steptoe et al. 2013). Written informed consent was obtained from all participants. Data were collected biennially through face‐to‐face computer‐assisted personal interviews and self‐completion questionnaires. Data from Waves 4 (2008–2009), 6 (2012–2013), and 8 (2016–2017) were used for cross‐sectional and prospective analyses, with Wave 4 considered the baseline assessment for prospective analyses and consistent definitions applied to all variables across analyses.

**FIGURE 1:**
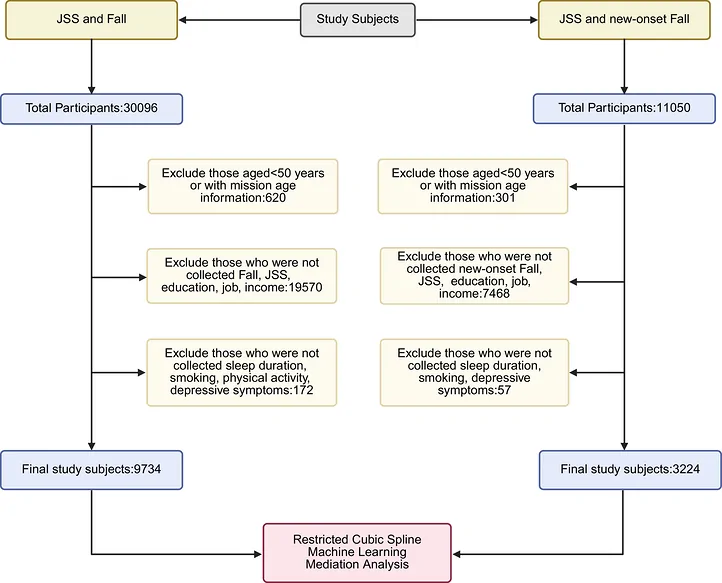
Flow chart of study population.

Participants aged s50 years with complete demographic, JSS, and outcome data were included. The cross‐sectional analysis primarily utilized data from Wave 4. To maximize statistical power and ensure a robust sample size, we employed a pooled cross‐sectional approach by additionally including participants from the refreshment cohorts newly recruited in Waves 6 and 8. To maintain the assumption of independent observations, duplicate participant IDs across different waves were removed, ensuring that only the first available observation for each unique individual was included in the cross‐sectional dataset. The baseline sample included 30,096 participants, of whom 620 aged < 50 years and 19,742 who lacked data for other key variables were excluded. For the prospective analysis, we included participants who had not experienced falls at baseline (Wave 4). We excluded 301 participants aged < 50 years and 7,525 with missing data on other key variables. After applying the inclusion and exclusion criteria, 9,734 and 3,224 participants were included in the cross‐sectional and prospective analyses, respectively (Figure 1). The present analyses were based on publicly available data approved by the relevant review committees and were conducted in accordance with the principles of the Declaration of Helsinki (World Medical Association 2013).

### 2.2 Assessment of Exposure

Sleep quality was evaluated using four items: difficulty falling asleep, repeated awakenings during the night, tiredness after waking, and overall dissatisfaction with sleep. This measure was adapted from the JSS, a concise and commonly used instrument for assessing sleep quality and sleep‐related health (Jenkins et al. 1988; Song et al. 2023). The first three items corresponded to three JSS questions: trouble falling asleep, waking several times per night, and waking up tired. Responses were scored as follows: not at all in the past month = 1, less than once weekly = 2, 1–2 times weekly = 3, and ≥3 times weekly = 4. The fourth item measured perceived overall sleep quality and was coded as very good = 1, good = 2, poor = 3, and very poor = 4. The four item scores were summed to obtain a total sleep quality score ranging from 4 to 16, with higher values reflecting poorer sleep quality. Participants were then classified into three categories: good sleep quality (4 ≤ score < 8), intermediate sleep quality (8 ≤ score < 12), and poor sleep quality (12 ≤ score ≤ 16).

Sleep duration was assessed using a self‐reported ELSA questionnaire item on participants’ average nightly hours of sleep. Sleep duration was primarily treated as a continuous variable in regression analyses. For further exploration of potential nonlinear associations, sleep duration was additionally categorized into quartiles according to its distribution, with the lowest quartile defined as the reference category.

### 2.3 Assessment of Outcome

Self‐reported falls were recorded as a binary variable. In each wave, participants were asked whether they had experienced a fall within the past two years. In the cross‐sectional analysis, falls were assessed at a single time point for each participant. By pooling the baseline data from Wave 4 and the refreshment cohorts from Waves 6 and 8 (with duplicate IDs strictly removed), the cross‐sectional outcome represents the prevalence of falls among unique individuals. In the prospective cohort analysis, new‐onset falls were defined as at least one fall reported during follow‐up in Wave 6 or Wave 8 among participants who reported no falls at baseline (Wave 4). New‐onset falls reported in Waves 6 and 8 were combined into a single outcome variable to represent cumulative fall occurrence over the follow‐up period. The cross‐sectional analysis examined the association between the JSS score and falls at a single time point, whereas the prospective cohort analysis captured new‐onset fall events during follow‐up.

### 2.4 Assessment of Covariates

Covariates were selected a priori based on previous literature and included sociodemographic characteristics, lifestyle factors, and chronic conditions. Sociodemographic variables included age, sex, occupation, income, educational level, and marital status. Occupation was classified according to the National Statistics Socio‐Economic Classification (NS‐SEC) into managerial and professional occupations, intermediate occupations, routine and manual occupations, and not in work. Total annual personal income was categorized into quintiles. Educational attainment was grouped as below high school, high school, college or above, and other. Marital status was categorized as married or partnered and other marital statuses (divorced, separated, widowed, or unmarried).

Lifestyle variables included physical activity, smoking, and drinking. Physical activity was measured by asking participants how frequently they performed vigorous, moderate, and light activities. For each intensity level, response options were hardly ever or never, one to three times per month, once weekly, and several times weekly. Each activity category was dichotomized into ≤ once per week, including hardly ever or never, one to three times per month, and once weekly, or > once per week, corresponding to several times weekly. Participants were then classified according to whether they undertook moderate or vigorous physical activity more than once per week. Smoking was based on self‐reported smoking history, with current smokers defined as smokers. Drinking was assessed using self‐reported information from each wave. Participants were asked whether they consumed alcohol and were categorized as drinkers or non‐drinkers according to their alcohol intake during the previous seven days.

Chronic health conditions included self‐reported hypertension, diabetes, high cholesterol, heart disease, chronic lung disease, and cancer. Depressive symptoms were measured with the eight‐item Centre for Epidemiological Studies Depression Scale (CES‐D) (Radloff 1977). To reduce potential overlap with the assessment of sleep quality, the sleep‐related item (“my sleep was restless”) was removed from the scale (Bowman et al. 2021). Consequently, the modified CES‐D score ranged between 0 and 7, with higher values indicating more severe depressive symptoms (Poole and Jackowska 2018).

Medication use was assessed during nurse visits at Wave 6. Participants were asked to report their currently prescribed medications, which were recorded by trained nurses and coded according to the British National Formulary. The use of hypnotics or sedatives was identified based on the medication codes and was included as a covariate in the supplementary analyses using Wave 6 data. Specifically, hypnotic or sedative use was adjusted for in multivariable models examining the cross‐sectional associations at Wave 6 and the prospective associations between Wave 6 and Wave 8.

### 2.5 Statistical Analysis

For descriptive statistics, continuous variables were presented as mean ± standard deviation (SD) or interquartile range, and as percentages for categorical variables. Because the ELSA database recorded only whether participants experienced falls during each follow‐up period (Wave 6 or Wave 8), without exact dates of fall occurrence, survival time could not be accurately calculated. Therefore, logistic regression was used in both the cross‐sectional and prospective cohort analyses to examine the associations of sleep quality with falls and new‐onset falls. Odds ratios (ORs) and 95% confidence intervals (CIs) were estimated using four sequential models. Model 1 was adjusted for age, sex, job, income, sleep duration, education, and marital status. Model 2 was additionally adjusted for lifestyle factors, including physical activity, smoking, and drinking. Model 3 further included chronic conditions, namely hypertension, diabetes, high cholesterol, heart disease, chronic lung disease, and cancer, as well as depressive symptoms. RCS analyses were used to visualize potential dose–response relationships between JSS score and the risks of falls and new‐onset falls.

To determine the main factors related to falls, LASSO regression was combined with the Boruta algorithm. LASSO regression was conducted with the “glmnet” package, which performs variable selection by applying a penalty to the absolute magnitude of regression coefficients (Tibshirani 2018; Friedman et al. 2010). The optimal λ parameter was selected through ten‐fold cross‐validation using the cv.glmnet function. The Boruta algorithm was then used to estimate feature relevance by comparing observed variables with randomly generated shadow variables. Feature importance was quantified using the random forest‐based getImpRfZ method (Kursa and Rudnicki 2010; Degenhardt et al. 2019). LASSO regression was implemented with the mixing parameter α set to 1. The Boruta algorithm was performed with a significance threshold of *p* < 0.01 and a maximum of 100 iterations to identify robustly associated factors.

For the mediation analysis, the R package “mediation” was used to evaluate whether key variables mediated the associations between JSS scores and falls or new‐onset falls (Imai et al. 2010; Tingley et al. 2014). All mediation models were fitted using the fully adjusted set of covariates. Mediation was assessed by examining the total effect of JSS scores on each outcome, the association between JSS scores and each mediator, and the association between each mediator and the outcome. Bootstrap resampling with 1,000 replications was used to calculate 95% CIs for the indirect and total effects. Evidence of mediation was considered present when the 95% CI for the indirect effect did not include zero (Preacher and Hayes 2008).

Additional subgroup analyses were performed to examine whether the associations of sleep quality with falls and new‐onset falls differed by age, sex, job, and other relevant characteristics. Sensitivity analyses were conducted after excluding participants who reported sleeping <6 or >9 h per night, thereby testing whether the associations between poor sleep quality and falls or new‐onset falls remained robust among individuals with normal sleep duration. All analyses were conducted using R, with statistical significance set at *p* < 0.05.

## 3 Results

### 3.1 Baseline Characteristics

A total of 9,734 participants were included in the cross‐sectional analysis, of whom 2,446 reported falls. Participants who reported falls were significantly older on average than those who did not. Women and individuals classified as “not in work” accounted for higher proportions of the fall group. Compared with the non‐fall group, participants with falls had significantly higher JSS scores, a lower proportion of good sleep quality, and shorter sleep duration. They also had relatively lower educational attainment, a lower proportion of being married or partnered, and were less likely to engage in physical activity or drinking. Regarding chronic conditions, the fall group had higher proportions of hypertension, diabetes, heart disease, and chronic lung disease. Depressive symptoms were also significantly higher among participants with falls. In contrast, income, smoking, high cholesterol, and cancer did not differ significantly between groups.

In the analysis of new‐onset falls, participants were grouped according to whether they reported a new fall during follow‐up. Among the 3,224 included participants, 1,452 reported falls during follow‐up. The distributions of age, sex, job, income, JSS score, sleep quality, education, marital status, physical activity, smoking, drinking, high cholesterol, heart disease, cancer, and depressive symptoms in the new‐onset fall group were similar to those observed in the cross‐sectional analysis. However, sleep duration, hypertension, diabetes, and chronic lung disease showed no significant group differences (Table 1).

**TABLE 1:** Baseline characteristics of the populations with fall and new‐onset fall.

Variables	Fall				New‐onset Fall			
	Total (*n* = 9734)	No (*n* = 7288)	Yes (*n* = 2446)	*p‐*value	Total (*n* = 3224)	No (*n* = 1772)	Yes (*n* = 1452)	*p‐*value
**Age**	68.11 ± 7.76	67.60 ± 7.39	69.63 ± 8.60	< 0.0001	68.32 ± 6.56	67.23 ± 5.76	69.66 ± 7.19	< 0.0001
**Sex**				< 0.0001				< 0.0001
female	5289 (54.34)	3834 (52.61)	1455 (59.48)		1714 (53.16)	881 (49.72)	833 (57.37)	
male	4445 (45.66)	3454 (47.39)	991 (40.52)		1510 (46.84)	891 (50.28)	619 (42.63)	
**Job**				< 0.0001				< 0.0001
managerial and professional occupations	999 (10.26)	775 (10.63)	224 (9.16)		275 (8.53)	172 (9.71)	103 (7.09)	
intermediate occupations	803 (8.25)	664 (9.11)	139 (5.68)		252 (7.82)	158 (8.92)	94 (6.47)	
routine and manual occupations	1009 (10.37)	825 (11.32)	184 (7.52)		300 (9.31)	182 (10.27)	118 (8.13)	
not in work	6923 (71.12)	5024 (68.94)	1899 (77.64)		2397 (74.35)	1260 (71.11)	1137 (78.31)	
**Income**				0.11				0.33
Q1	1949 (20.02)	1474 (20.23)	475 (19.42)		646 (20.04)	359 (20.26)	287 (19.77)	
Q2	1951 (20.04)	1442 (19.79)	509 (20.81)		644 (19.98)	338 (19.07)	306 (21.07)	
Q3	1941 (19.94)	1428 (19.59)	513 (20.97)		644 (19.98)	345 (19.47)	299 (20.59)	
Q4	1946 (19.99)	1448 (19.87)	498 (20.36)		645 (20.01)	356 (20.09)	289 (19.90)	
Q5	1947 (20.00)	1496 (20.53)	451 (18.44)		645 (20.01)	374 (21.11)	271 (18.66)	
**JSS**	8.81 ± 3.16	8.59 ± 3.07	9.49 ± 3.31	< 0.0001	8.53 ± 3.01	8.31 ± 2.97	8.81 ± 3.04	< 0.0001
**Sleep quality**				< 0.0001				< 0.0001
Poor	1987 (20.41)	1315 (18.04)	672 (27.47)		547 (16.97)	265 (14.95)	282 (19.42)	
Intermediate	4171 (42.85)	3130 (42.95)	1041 (42.56)		1412 (43.80)	755 (42.61)	657 (45.25)	
Good	3576 (36.74)	2843 (39.01)	733 (29.97)		1265 (39.24)	752 (42.44)	513 (35.33)	
**Sleep duration**	6.86 ± 1.35	6.89 ± 1.28	6.76 ± 1.52	< 0.001	6.91 ± 1.25	6.92 ± 1.21	6.88 ± 1.29	0.35
**Education**				< 0.01				< 0.01
Below high school	3277 (33.67)	2386 (32.74)	891 (36.43)		1062 (32.94)	551 (31.09)	511 (35.19)	
High school	2477 (25.45)	1873 (25.70)	604 (24.69)		831 (25.78)	484 (27.31)	347 (23.90)	
College or above	2894 (29.73)	2219 (30.45)	675 (27.60)		1026 (31.82)	588 (33.18)	438 (30.17)	
Other	1086 (11.16)	810 (11.11)	276 (11.28)		305 (9.46)	149 (8.41)	156 (10.74)	
**Marital status**				< 0.0001				< 0.0001
Married or partnered	6848 (70.35)	5306 (72.80)	1542 (63.04)		2314 (71.77)	1327 (74.89)	987 (67.98)	
Other marital status	2886 (29.65)	1982 (27.20)	904 (36.96)		910 (28.23)	445 (25.11)	465 (32.02)	
**Physical activity**				< 0.0001				< 0.001
No	3648 (37.48)	2552 (35.02)	1096 (44.81)		1026 (31.82)	519 (29.29)	507 (34.92)	
Yes	6086 (62.52)	4736 (64.98)	1350 (55.19)		2198 (68.18)	1253 (70.71)	945 (65.08)	
**Smoking**				0.53				0.91
No	8539 (87.72)	6384 (87.60)	2155 (88.10)		2890 (89.64)	1587 (89.56)	1303 (89.74)	
Yes	1195 (12.28)	904 (12.40)	291 (11.90)		334 (10.36)	185 (10.44)	149 (10.26)	
**Drinking**				< 0.0001				< 0.01
No	1836 (18.86)	1365 (18.73)	471 (19.26)		574 (17.80)	311 (17.55)	263 (18.11)	
Yes	5574 (57.26)	4283 (58.77)	1291 (52.78)		2027 (62.87)	1151 (64.95)	876 (60.33)	
Don't know	2324 (23.88)	1640 (22.50)	684 (27.96)		623 (19.32)	310 (17.49)	313 (21.56)	
**Hypertension**				< 0.0001				0.21
No	4427 (45.48)	3399 (46.64)	1028 (42.03)		1428 (44.29)	803 (45.32)	625 (43.04)	
Yes	5307 (54.52)	3889 (53.36)	1418 (57.97)		1796 (55.71)	969 (54.68)	827 (56.96)	
**Diabetes**				< 0.001				0.24
No	8498 (87.30)	6417 (88.05)	2081 (85.08)		2847 (88.31)	1576 (88.94)	1271 (87.53)	
Yes	1236 (12.70)	871 (11.95)	365 (14.92)		377 (11.69)	196 (11.06)	181 (12.47)	
**High cholesterol**				0.56				0.90
No	6397 (65.72)	4802 (65.89)	1595 (65.21)		2158 (66.94)	1184 (66.82)	974 (67.08)	
Yes	3337 (34.28)	2486 (34.11)	851 (34.79)		1066 (33.06)	588 (33.18)	478 (32.92)	
**Heart disease**				< 0.0001				< 0.0001
No	7598 (78.06)	5845 (80.20)	1753 (71.67)		2625 (81.42)	1497 (84.48)	1128 (77.69)	
Yes	2136 (21.94)	1443 (19.80)	693 (28.33)		599 (18.58)	275 (15.52)	324 (22.31)	
**Chronic lung disease**				< 0.001				0.22
No	9142 (93.92)	6879 (94.39)	2263 (92.52)		3079 (95.50)	1700 (95.94)	1379 (94.97)	
Yes	592 (6.08)	409 (5.61)	183 (7.48)		145 (4.50)	72 (4.06)	73 (5.03)	
**Cancer**				0.13				0.26
No	8839 (90.81)	6637 (91.07)	2202 (90.02)		2958 (91.75)	1635 (92.27)	1323 (91.12)	
Yes	895 (9.19)	651 (8.93)	244 (9.98)		266 (8.25)	137 (7.73)	129 (8.88)	
**Depressive symptoms**	1.02 ± 1.66	0.86 ± 1.53	1.48 ± 1.93	< 0.0001	0.81 ± 1.48	0.65 ± 1.34	1.00 ± 1.62	< 0.0001

*Note*: All values were presented as Mean ± SD, or counts (proportion).

To evaluate the potential influence of attrition bias on the prospective analysis, baseline characteristics were compared between participants included in the follow‐up analysis and those lost to follow‐up. Several differences were observed between the two groups: participants retained for follow‐up were older and had higher prevalences of hypertension, diabetes, high cholesterol, heart disease, and cancer. In addition, retained participants had a lower prevalence of poor sleep quality (Table S1).

### 3.2 Relationship Between Sleep Quality and Falls

**FIGURE 2:**
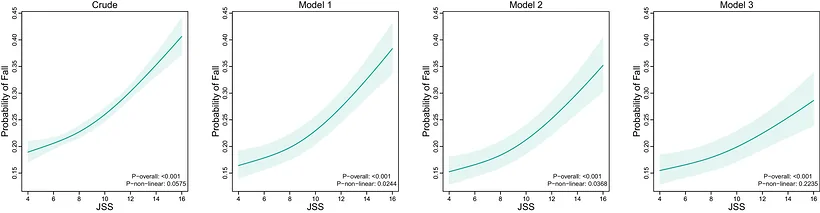
Association between JSS and fall based on RCS analysis across different models. The x‐axis represents the JSS, and the y‐axis represents the probability.

In the cross‐sectional analysis, the JSS score was significantly and positively associated with fall risk, and the association remained stable across successive models. In the crude model, each increase in JSS score corresponded to greater odds of falls (OR = 1.09, 95% CI: 1.08–1.11, *p* < 0.0001), suggesting that poorer sleep quality was associated with greater fall risk. After adjustment for age, sex, job, income, sleep duration, education, and marital status, the association persisted (OR = 1.10, 95% CI: 1.08–1.12, *p* < 0.0001). Additional adjustment for physical activity, smoking, and drinking in Model 2 produced similar results (OR = 1.09, 95% CI: 1.08–1.11, *p* < 0.0001). In Model 3, after additional adjustment for chronic conditions, including hypertension, diabetes, high cholesterol, heart disease, chronic lung disease, cancer, and depressive symptoms, the association between the JSS score and fall risk remained robust (OR = 1.07, 95% CI: 1.05–1.09, *p* < 0.0001). Regression analyses based on JSS score categories showed similar results. Relative to participants with poor sleep quality, those reporting moderate or good sleep quality had lower odds of falls, and these inverse associations remained robust across models (Table 2). RCS curves further visualized the association between the JSS score and fall risk in these models (Figure 2). After adjustment for covariates, the JSS score showed a significant linear positive association with fall risk, indicating that fall risk increased significantly as sleep quality worsened (*p*‐overall < 0.001, *p*‐nonlinear > 0.05).

**TABLE 2:** Multivariate logistic regression analysis of sleep quality and fall.

Variable	Crude model		Model 1		Model 2		Model 3	
	OR(95%CI)	*p‐*value	OR(95%CI)	*p‐*value	OR(95%CI)	*p‐*value	OR(95%CI)	*p‐*value
JSS	1.09 (1.08,1.11)	< 0.0001	1.10 (1.08,1.12)	< 0.0001	1.09 (1.08,1.11)	< 0.0001	1.07 (1.05,1.09)	< 0.0001
Sleep quality								
Poor	1(Ref)		1(Ref)		1(Ref)		1(Ref)	
Intermediate	0.65 (0.58,0.73)	< 0.0001	0.65 (0.57,0.74)	< 0.0001	0.67 (0.59,0.76)	< 0.0001	0.76 (0.67,0.87)	< 0.0001
Good	0.50 (0.45,0.57)	< 0.0001	0.51 (0.44,0.58)	< 0.0001	0.52 (0.45,0.60)	< 0.0001	0.65 (0.56,0.75)	< 0.0001
Trend test		< 0.0001		< 0.0001		< 0.0001		< 0.0001

Crude model: Unadjusted.

Model 1: Adjusted for age, sex, job, income, sleep duration, education, and marital status.

Model 2: Adjusted for age, sex, job, income, sleep duration, education, marital status, physical activity, smoking, and drinking.

Model 3: Adjusted for age, sex, job, income, sleep duration, education, marital status, physical activity, smoking, drinking, hypertension, diabetes, high cholesterol, heart disease, chronic lung disease, cancer, and depressive symptoms.

Abbreviations: CI, confidence interval; OR, odds ratio; Ref: reference.

**FIGURE 3:**
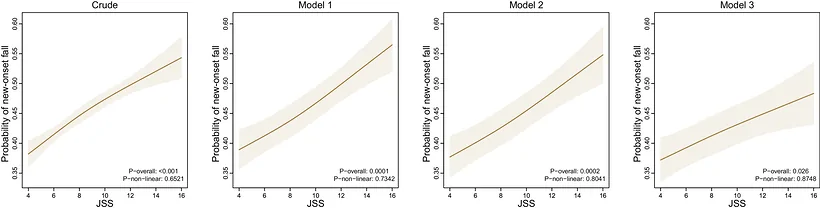
Association between JSS and new‐onset fall based on RCS analysis across different models. The x‐axis represents the JSS, and the y‐axis represents the probability.

In the prospective analysis, a comparable pattern was identified. The JSS score was positively associated with new‐onset falls. After covariate adjustment, each 1‐point increase in the JSS score corresponded to a 4% increase in the odds of new‐onset falls (OR = 1.04, 95% CI: 1.01–1.07, *p* < 0.05). When sleep quality was examined categorically, participants with good sleep quality had significantly lower odds of new‐onset falls than those with poor sleep quality (Table 3). RCS analysis yielded results consistent with the cross‐sectional findings (Figure 3). In the adjusted model, the JSS score demonstrated a positive linear relationship with new‐onset fall risk, indicating that fall risk increased as sleep quality worsened (*p*‐overall < 0.001, *p*‐nonlinear > 0.05).

**TABLE 3:** Multivariate logistic regression analysis of sleep quality and new‐onset fall.

Variable	Crude model		Model 1		Model 2		Model 3	
	OR(95%CI)	*p‐*value	OR(95%CI)	*p‐*value	OR(95%CI)	*p‐*value	OR(95%CI)	*p‐*value
JSS	1.06 (1.03,1.08)	< 0.0001	1.06 (1.03,1.09)	< 0.0001	1.06 (1.03,1.09)	< 0.0001	1.04 (1.01,1.07)	0.01
Sleep quality								
Poor	1(Ref)		1(Ref)		1(Ref)		1(Ref)	
Intermediate	0.82 (0.67,1.00)	0.05	0.77 (0.62,0.95)	0.02	0.78 (0.62,0.96)	0.02	0.86 (0.68,1.07)	0.17
Good	0.64 (0.52,0.78)	< 0.0001	0.62 (0.50,0.79)	< 0.0001	0.63 (0.50,0.79)	< 0.0001	0.73 (0.57,0.93)	0.01
Trend test		< 0.0001		< 0.0001		< 0.0001		0.01

*Note*: Crude model: Unadjusted.

Model 1: Adjusted for age, sex, job, income, sleep duration, education, and marital status.

Model 2: Adjusted for age, sex, job, income, sleep duration, education, marital status, physical activity, smoking, and drinking.

Model 3: Adjusted for age, sex, job, income, sleep duration, education, marital status, physical activity, smoking, drinking, hypertension, diabetes, high cholesterol, heart disease, chronic lung disease, cancer, and depressive symptoms.

Abbreviations: CI, confidence interval; OR, odds ratio; Ref: reference.

### 3.3 Selection of Variables

Key variables were identified using both LASSO regression and the Boruta algorithm. In the cross‐sectional analysis, the variables selected by LASSO regression under the 1‐SE criterion included heart disease, depressive symptoms, JSS, age, and marital status (Figure S1A–S1B). In the Boruta analysis, variables identified as important features were shown in green, whereas rejected features were shown in red (Figure S1C–S1D). Finally, heart disease, depressive symptoms, JSS, age, and marital status were identified as key variables in the cross‐sectional analysis (Figure S1E).

In the prospective analysis, the key variables selected by LASSO regression included sex, heart disease, depressive symptoms, JSS, and age (Figure S2A–S2B). The Boruta algorithm further confirmed heart disease, depressive symptoms, JSS, and age as key variables (Figure S2C–S2D). Ultimately, the key variables identified in the prospective analysis were heart disease, depressive symptoms, JSS, and age (Figure S2E).

### 3.4 Mediating Effects

**FIGURE 4:**
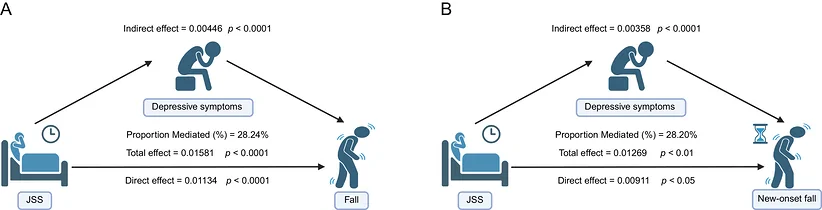
Mediation analyses of depressive symptoms in the association between JSS, fall (A), and new‐onset fall (B).

Mediation analysis further clarified the potential roles of key variables in the association between the JSS score and fall risk. Although age and marital status were identified as important variables by the machine learning algorithms, age was not considered a mediator because it is intrinsically time‐dependent and increases irreversibly. Marital status was also not included as a mediator because it primarily reflects an individual's social background. Therefore, neither variable was examined in the mediation analysis (Tables S2–S3). In the cross‐sectional analysis, heart disease and depressive symptoms both showed significant mediating effects in the association between the JSS score and falls, with depressive symptoms showing the highest proportion mediated, at 28.24% (Figure 4A). Similar findings were observed in the prospective analysis, in which the proportion mediated by depressive symptoms was 28.20% (Figure 4B). In both the cross‐sectional and prospective analyses, depressive symptoms showed a significant partial mediating effect.

### 3.5 Subgroup Analyses

**FIGURE 5:**
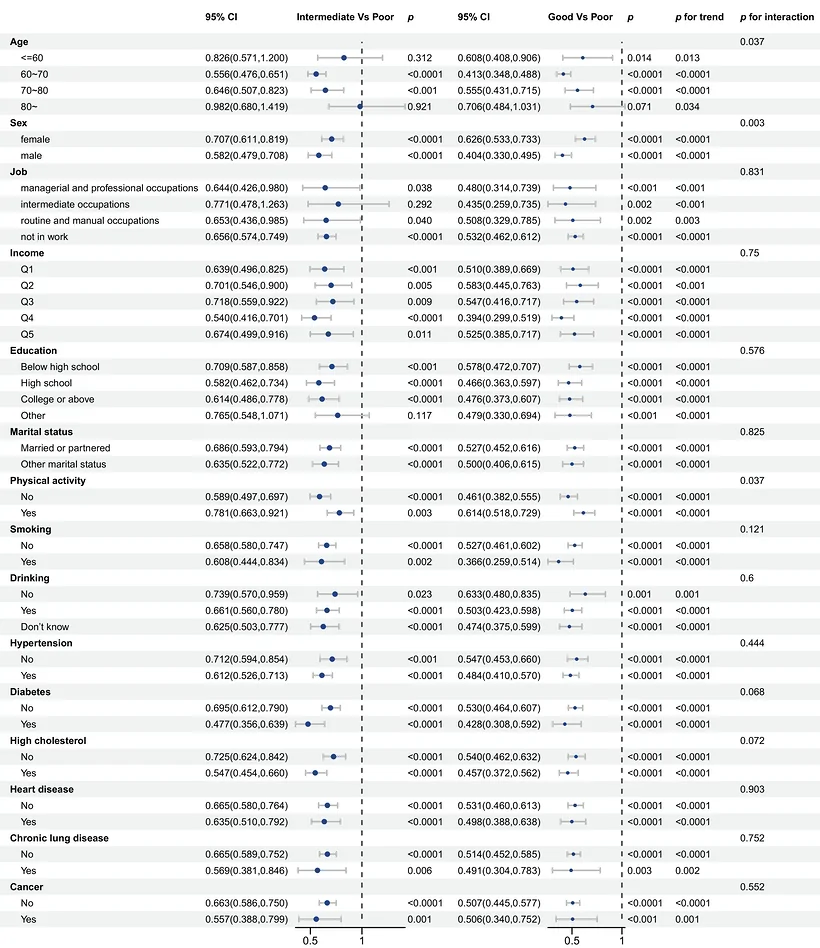
Forest plot of logistic regression analysis based on the subgroup that comprises JSS and fall.

**FIGURE 6:**
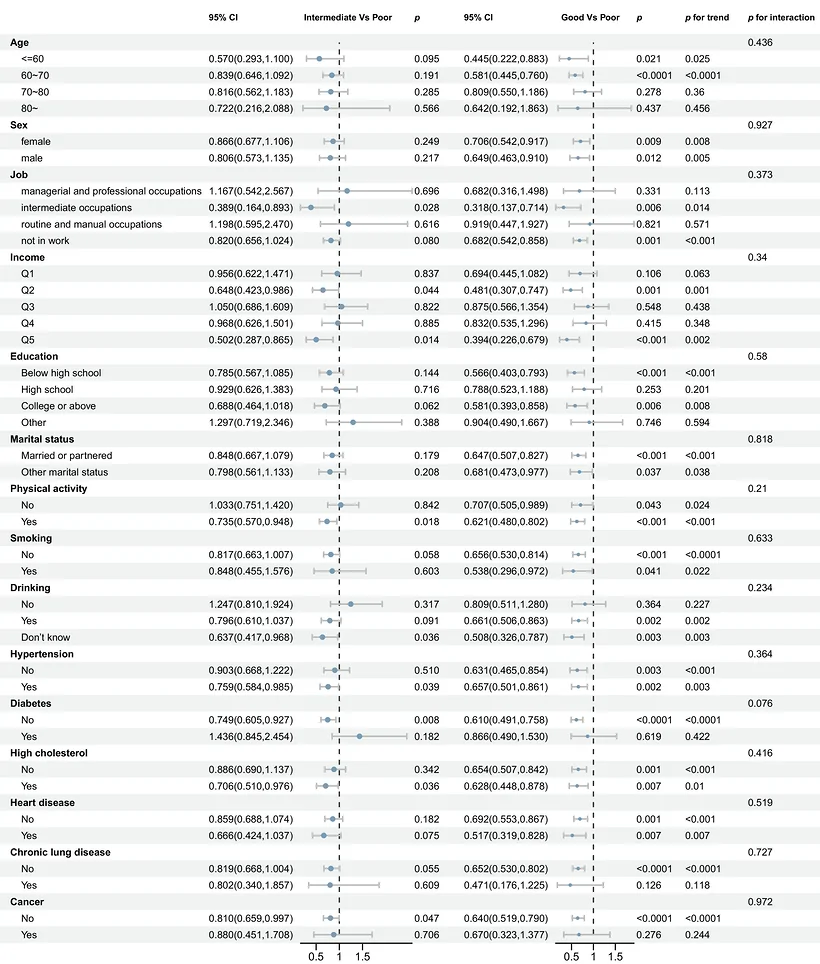
Forest plot of logistic regression analysis based on the subgroup that comprises JSS and new‐onset fall.

In the cross‐sectional analysis, significant interactions were found for age, sex, and physical activity with respect to the association between sleep quality and fall risk. The inverse association between better sleep quality and fall risk was more pronounced among adults aged 60–70 years, men, and participants reporting less frequent physical activity. Although the strength of the association varied across some subgroups, the overall pattern was consistent: better sleep quality was associated with a lower likelihood of falls compared with poorer sleep quality (Figure 5). In the prospective analysis, no statistically significant subgroup interactions were observed. Nevertheless, better sleep quality remained associated with a lower overall likelihood of new‐onset falls compared with poor sleep quality (Figure 6).

### 3.6 Sensitivity Analyses

To assess whether the association of sleep quality with fall risk persisted beyond the influence of abnormal sleep duration, we performed a sensitivity analysis after excluding participants who reported short (<6 h) or long (>9 h) sleep duration. In this subgroup, the association remained robust. After adjustment for relevant covariates, participants with intermediate sleep quality had lower odds of falls than those with poor sleep quality (OR = 0.72, 95% CI: 0.62–0.84, *p* < 0.0001), with an even greater reduction among participants reporting good sleep quality (OR = 0.60, 95% CI: 0.50–0.71, *p* < 0.0001). Accordingly, worse sleep quality corresponded to greater fall risk (Table S4). RCS analysis further showed that fall risk increased as JSS scores rose within this subgroup (Figure S3). The same sensitivity analysis was conducted for new‐onset falls (Table S5). In the fully adjusted model, participants with good sleep quality had 32% lower odds of new‐onset falls than those with poor sleep quality (OR = 0.68, 95% CI: 0.51–0.90, *p* < 0.05). The covariate‐adjusted RCS results were consistent with the main analyses (Figure S4). Together, these results indicate that sleep quality remains meaningfully associated with fall risk even when sleep duration is within the normal range.

Table S6 presents the association between sleep duration and fall risk. After covariate adjustment, sleep duration was not significantly associated with falls. When sleep duration was divided into quartiles, the results were generally consistent across models. Compared with the lowest quartile of sleep duration (Hours Q1), participants in Hours Q2 had a lower risk of falls, whereas Hours Q3 and Q4 showed no significant associations. The RCS curve suggested a U‐shaped association between sleep duration and falls (Figure S5). In the prospective analysis, sleep duration was also not significantly associated with new‐onset falls across all models (Table S7).

To assess the potential influence of sedative/hypnotic medication use, medication use at Wave 6 was additionally adjusted for as a covariate in sensitivity analyses. In the Wave 6 cross‐sectional analysis, the positive association between JSS scores and fall risk remained robust after adjustment for sedative/hypnotic medication use (OR = 1.05, 95% CI: 1.02–1.07, *p* < 0.0001). Analyses based on JSS quartiles yielded consistent results (Table S8). In the prospective analysis using data from Wave 6 to Wave 8, a similar association pattern was observed between JSS scores and the risk of new‐onset falls (Table S9). RCS analyses further confirmed the robustness of the main findings (Figures S6–S7). In addition, the mediating effect of depressive symptoms remained significant, accounting for 34.06% and 21.05% of the total association, respectively (Figure S8). Furthermore, sensitivity analyses were repeated after excluding participants who reported sedative/hypnotic medication use. The associations between JSS scores and both fall and new‐onset fall remained consistent in regression models (Tables S10–S11). The dose–response patterns identified by RCS analyses were also comparable with those observed in the primary analyses (Figures S9–S10). After excluding sedative/hypnotic medication users, depressive symptoms continued to show a significant mediating effect, accounting for 33.90% and 18.93% of the total association, respectively (Figure S11).

## 4 Discussion

This study found that higher JSS scores were significantly associated with greater risks of both falls and new‐onset falls, suggesting that poorer sleep quality was linked to increased fall risk in both analyses. These associations remained robust after adjustment for covariates. Machine‐learning‐based variable selection identified heart disease, depressive symptoms, JSS score, and age as key variables across different time frames, further highlighting their relevance to fall risk. Mediation analysis showed that depressive symptoms partially mediated these associations, with mediation proportions of 28.24% for falls and 28.20% for new‐onset falls. These findings suggest that depressive symptoms may represent an important intermediate factor in the association between sleep quality and fall risk in older adults and may offer a new perspective for designing comprehensive interventions. In the cross‐sectional analysis based on sleep duration, after adjustment for all covariates, the RCS curve showed that the lowest fall risk occurred at 6.85 h of sleep. Although a U‐shaped pattern relating sleep duration to falls was evident in the cross‐sectional analysis, this association may be influenced by multiple factors during longer‐term follow‐up. Overall, this study further emphasizes the importance of incorporating both sleep quality and mental health into fall risk evaluation and prevention planning, thereby providing new evidence to inform comprehensive and modifiable interventions for fall prevention.

Falls can result in severe outcomes, including traumatic brain injuries and hip fractures, and represent a major global cause of mortality from unintentional injuries, placing considerable pressure on families and society. Sleep is a basic physiological process and has increasingly been recognized as important for physical function and behavioral safety. Growing evidence suggests that poor sleep quality is associated with greater fall risk. A Korean community‐based study using the PSQI found higher odds of falling among individuals with poor sleep quality (Lee et al. 2021). Similarly, the Outcomes of Sleep Disorders in Older Men study reported that subjective and objectively measured sleep disturbances were associated with increased fall risk in older men (Stone et al. 2014). Consistent with these studies, our analyses based on the JSS showed that poorer sleep quality was associated with higher odds of falls in both cross‐sectional and prospective settings. Sleep duration has also been proposed as a relevant factor in fall risk. Previous cross‐sectional evidence indicated that sleeping ≤5 h or ≥8 h was associated with a higher risk of fall‐related injuries, which partly aligns with our results. The present cross‐sectional findings indicated a nonlinear, U‐shaped association between sleep duration and fall risk, with the lowest risk observed at approximately 7 h after covariate adjustment. In addition, a prospective study using CHARLS data reported a significantly increased fall risk among individuals reporting short sleep duration, whereas no significant association was observed for long sleep duration (Bai et al. 2026). By contrast, a prospective study found that both shorter and longer sleep durations were independently associated with a higher risk of recurrent falls (Cauley et al. 2019). Sleep duration not only reflects sleep‐related behaviors but may also serve as an indicator of underlying health status. Previous studies have demonstrated that longer sleep duration is associated with an increased risk of frailty among older adults (Baniak et al. 2018). Rather than simply reflecting an isolated sleep behavior, prolonged sleep duration may represent a potential marker of reduced physiological reserve and increased health vulnerability (Nakakubo et al. 2018). Frailty is a multidimensional syndrome characterized by impaired physiological recovery capacity, reduced muscle strength, and other functional declines, all of which are closely linked to an elevated risk of falls. Moreover, older adults with multimorbidity or functional limitations may experience prolonged sleep or extended time spent resting due to reduced physiological reserve and mobility limitations; meanwhile, these underlying health conditions may independently contribute to an increased risk of falls (Deandrea et al. 2010). In our prospective analysis, however, sleep duration was not significantly related to new‐onset falls. This inconsistency may reflect differences in study populations, fall definitions, and within‐person variation in sleep duration during follow‐up. Importantly, the associations among sleep quality, sleep duration, and depression are likely bidirectional rather than one‐way. A systematic review reported a longitudinal reciprocal relationship between sleep disturbance and depression (Alvaro et al. 2013), which is supported by another prospective study of 24,715 participants (Sivertsen et al. 2012). These findings indicate that sleep problems and depression may reinforce each other. Persistent poor sleep may weaken emotional regulation and increase susceptibility to depression, while depressive symptoms may impair sleep onset and maintenance through pathways such as neuroendocrine dysfunction and circadian rhythm disruption (Fang et al. 2019).

Biological pathways linking poor sleep quality to elevated fall risk have garnered increasing attention. Evidence from experimental studies suggests that sleep restriction may adversely affect neuromuscular and metabolic function. For instance, Nicholas J. Saner and colleagues, using muscle biopsies, found that sleep restriction was associated with a significant decrease in myofibrillar protein synthesis, potentially contributing to muscle mass loss (Saner et al. 2020). A randomized crossover study similarly demonstrated that sleep deprivation suppresses muscle protein synthesis and may promote a catabolic environment (Lamon et al. 2021). Sleep restriction has also been linked to reduced mitochondrial respiratory capacity and impaired glucose tolerance (Saner et al. 2021). Collectively, poor sleep quality may impair muscle repair and energy metabolism, manifesting clinically as reduced exercise tolerance, decreased joint flexibility, and diminished muscle strength. Fall‐related research and risk assessments have traditionally focused on physical function—particularly muscle strength, gait, and related performance‐based measures—and screening and intervention frameworks have largely centered on physical fitness assessments (Strini et al. 2021). However, growing evidence suggests that affective and psychological factors also play a crucial role in the occurrence and progression of falls. In this context, the mediating role of depressive symptoms identified in our study offers additional insights into the pathways linking sleep disturbances to fall risk. Multiple components of the JSS are closely linked to depressive symptoms. Difficulty initiating sleep, frequent nocturnal awakenings, and waking up tired are hallmark features of insomnia, which is highly comorbid with depression (Baglioni et al. 2011). Sleep fragmentation and circadian dysregulation may contribute to the onset or exacerbation of depressive symptoms through alterations in neuroendocrine function, emotion regulation, and stress responsivity, with the hypothalamic‐pituitary‐adrenal (HPA) axis potentially implicated (Riemann et al. 2010; Buckley and Schatzberg 2005). Depressive symptoms, in turn, may increase fall risk (Kvelde et al. 2015). Depression has been associated with executive dysfunction, psychomotor slowing, attentional deficits, and reduced motivation—domains integral to maintaining safe mobility (Iaboni and Flint 2013). Additionally, depression is often accompanied by reduced physical activity, lower muscle strength, slower gait speed, and impaired balance, and it may correlate with greater use of psychotropic medications; each of these factors may further increase vulnerability to falls (Pieruccini‐Faria et al. 2018; van Poelgeest et al. 2021). Taken together, depressive symptoms may serve as a key intermediary linking poor sleep quality to functional vulnerability and a higher likelihood of falls. The sleep status captured by the JSS may reflect not only sleep‐related disturbances but also broader affective and psychosocial distress. As a subjective measure, poor sleep quality may indicate early depressive symptoms or subclinical mood disturbances not fully captured by conventional covariate adjustments. It should be noted that depression and sleep are reciprocally related, and depressive symptoms may, in turn, exacerbate sleep disturbances. A structural equation modeling study found that depressive symptoms may impair sleep quality through rumination (Slavish and Graham‐Engeland 2014). In addition, dysfunction of the HPA axis and disruptions in cortisol rhythms may not only aggravate depressive symptoms but also maintain a heightened level of nocturnal arousal, thereby interfering with sleep initiation and maintenance and perpetuating a vicious cycle (Buckley and Schatzberg 2005). Overall, our findings suggest that depressive symptoms may be a key, potentially modifiable target for fall‐prevention interventions in individuals reporting poor sleep.

Given the close relationships among poor sleep quality, depressive symptoms, and elevated fall risk, improving sleep quality may be a valuable strategy for fall prevention. However, pharmacological treatments for sleep disorders, especially in older adults, are frequently linked to adverse effects such as cognitive impairment and increased fall risk (Full et al. 2025). Therefore, non‐pharmacological interventions have received growing interest as safer and more sustainable methods for enhancing sleep quality. Improvements in sleep quality may contribute to better physiological resilience and daytime functioning through enhanced physical recovery, reduced fatigue, and improved cognitive and neuromuscular performance (Corral‐Pérez et al. 2024; Casals et al. 2023). As adequate sleep is closely involved in maintaining muscle function, balance regulation, and postural control, better sleep quality may reduce functional impairments and increase resistance to fall‐related challenges, thereby lowering vulnerability to falls in older adults (Hand et al. 2025). Cognitive behavioral therapy for insomnia (CBT‐I) is a multicomponent intervention designed to address factors that perpetuate insomnia by combining cognitive therapy, education, and behavioral techniques, and it can be delivered through flexible formats. Clinical guidelines from the American College of Physicians endorse CBT‐I as the first‐line therapy for chronic insomnia (Qaseem et al. 2016). Evidence also indicates that interventions including CBT‐I produce greater improvements in sleep quality and depressive symptoms compared with standard care alone (Carney et al. 2017). In addition to CBT‐I, physical exercise may improve sleep quality and alleviate depressive symptoms. For instance, engaging in 12 weeks of moderate‐intensity aerobic exercise has been shown to significantly improve sleep quality and reduce depressive symptoms (Tseng et al. 2020), consistent with findings from a Chinese population‐based study of adults aged 60 years and older (Song and Yu 2019). Resistance training, yoga, and Tai Chi have likewise demonstrated beneficial effects on both depressive mood and sleep quality (Carcelén‐Fraile et al. 2024; Turmel et al. 2022; Chang et al. 2024). As research in this area grows, integrating multiple non‐pharmacological strategies may enable more individualized and effective clinical interventions.

This study leveraged data from the ELSA, a nationally representative population‐based cohort, in which the large sample size and comprehensive multidimensional health information provided a robust basis for evaluating the association between sleep quality and fall risk. Furthermore, candidate variables for mediation analysis were identified using LASSO regression and the Boruta algorithm, which may reduce potential bias arising from subjective variable selection. Notably, depressive symptoms were consistently identified as an important factor associated with falls across different study designs and variable selection approaches, supporting their potential role in the sleep–falls association. In addition, the association between sleep quality and fall risk was examined using both cross‐sectional and prospective cohort designs, and the robustness of the findings was further supported by multiple sensitivity analyses.

This study has several limitations that should be considered. First, fall events were assessed through self‐reports, which may have introduced recall bias. Declines in episodic memory and event retrieval abilities may compromise the accuracy of recalling previous falls among older adults. In particular, fall episodes that were not associated with apparent injuries, did not require medical attention, or occurred in the distant past may have been more susceptible to omission due to memory decline, limited event salience, or interference from other daily experiences, potentially resulting in underreporting of falls. Additionally, modeling falls as a binary variable limits the granularity of our findings, as it fails to distinguish between single and recurrent falls or between injurious and non‐injurious events. Second, objective measures of sleep quality were unavailable, and questionnaire‐based assessments may oversimplify the complexity of sleep as a physiological process. As a non‐invasive approach for objective sleep monitoring, wearable technologies (actigraphy) can capture multidimensional physiological and behavioral information, including sleep duration and sleep timing patterns (Saliba et al. 2026). Compared with traditional self‐reported questionnaires, wearable actigraphy provides a more objective assessment of habitual sleep characteristics in real‐world settings (Chee et al. 2025; Ghazi et al. 2024). Moreover, wearable actigraphy has emerged as a promising tool for fall risk assessment and has received increasing attention for identifying individuals at elevated risk of falls among older adults (Yang et al. 2019). Future research integrating objective sleep and activity monitoring data from wearable devices may enable more accurate characterization of sleep patterns and further clarify the longitudinal relationship between sleep disturbances and fall risk, thereby providing stronger evidence for precision fall risk stratification and personalized interventions in older adults. Third, attrition bias and selection bias may have influenced the prospective analyses. Although participants lost to follow‐up had a slightly higher proportion of poor sleep quality, the overall pattern of baseline differences did not suggest that individuals with worse sleep quality or poorer health status were systematically more likely to be lost to follow‐up. Furthermore, combining outcomes from Waves 6 and 8 without exact fall timing data limited our ability to capture the precise temporal sequence of events. Also, because exact time‐to‐event data were unavailable, we utilized logistic regression rather than survival analysis, which limited our ability to account for varying times at risk during the follow‐up period. Nevertheless, residual selection bias and potential survivor bias cannot be completely excluded. Fourth, as the study population comprised older adults from England, caution is needed when generalizing the findings to other populations. Fifth, despite adjustment for several important covariates, residual confounding from unmeasured factors influencing sleep and falls cannot be ruled out. Sixth, while machine learning approaches are effective for identifying relevant variables, the relative contribution of individual factors and their complex interactions remains to be fully understood. Finally, although depressive symptoms statistically mediated the associations between sleep quality and both falls and new‐onset falls, these findings should be interpreted with caution. The identified indirect effects do not establish causal pathways. Future research incorporating more objective assessments, larger sample sizes, and cross‐regional analyses is needed to further refine fall risk management strategies in older populations.

## 5 Conclusion

Using both cross‐sectional and prospective designs, this study showed that poor sleep quality, measured by the JSS, was significantly associated with higher risks of falls and new‐onset falls. These findings were generally stable in sensitivity and subgroup analyses. Depressive symptoms, identified as an important feature by LASSO regression and the Boruta algorithm, appeared to mediate the association. Future studies should assess whether interventions addressing both sleep quality and depressive symptoms can reduce fall risk and support the development of clinically applicable fall‐prevention strategies for adults aged 50 or older.

## Author Contributions

**Yiwei Li**: writing – original draft, conceptualization, validation, writing – review and editing. **Bowen Lu**: software, data curation, investigation. **Yifa Rong**: conceptualization, methodology, visualization, software. **Gang Li**: funding acquisition, formal analysis, supervision, project administration, resources. **Kai Jiang**: conceptualization, methodology. **Jiacheng Li**: software, data curation, investigation. **Xuezhen Liang**: software, data curation, investigation.

## Funding

We would like to acknowledge the following financial support: Key Technology Research and Development Program of Shandong Province (NO.2021CXGC010501); Leading Science and Technology Innovation Team (2024sdskctd‐02); and Natural Science Foundation of Shandong Province (ZR2022LZY003).

## Consent

All data used in this work are publicly available from studies with relevant participant consent and ethical approval.

## Conflicts of Interest

The authors declare no conflicts of interest.

## Supporting information




**Supplementary Figure**: brb371719‐sup‐0001‐FigureS1.pdf

**Supplementary Figure**: brb371719‐sup‐0002‐FigureS2.pdf

**Supplementary Figure**: brb371719‐sup‐0003‐FigureS3.pdf

**Supplementary Figure**: brb371719‐sup‐0004‐FigureS4.pdf

**Supplementary Figure**: brb371719‐sup‐0005‐FigureS5.pdf

**Supplementary Figure**: brb371719‐sup‐0006‐FigureS6.pdf

**Supplementary Figure**: brb371719‐sup‐0007‐FigureS7.pdf

**Supplementary Figure**: brb371719‐sup‐0008‐FigureS8.pdf

**Supplementary Figure**: brb371719‐sup‐0009‐FigureS9.pdf

**Supplementary Figure**: brb371719‐sup‐0010‐FigureS10.pdf

**Supplementary Figure**: brb371719‐sup‐0011‐FigureS11.pdf

**Supplementary Table**: brb371719‐sup‐00012‐TableS1.docx

**Supplementary Table**: brb371719‐sup‐00013‐TableS2.docx

**Supplementary Table**: brb371719‐sup‐00014‐TableS3.docx

**Supplementary Table**: brb371719‐sup‐00015‐TableS4.docx

**Supplementary Table**: brb371719‐sup‐00016‐TableS5.docx

**Supplementary Table**: brb371719‐sup‐00017‐TableS6.docx

**Supplementary Table**: brb371719‐sup‐00018‐TableS7.docx

**Supplementary Table**: brb371719‐sup‐00019‐TableS8.docx

**Supplementary Table**: brb371719‐sup‐00020‐TableS9.docx

**Supplementary Table**: brb371719‐sup‐00021‐TableS10.docx

**Supplementary Table**: brb371719‐sup‐00022‐TableS11.docx

## Data Availability

Publicly available datasets and materials were analyzed in this study.
