# Supplementary material for: Sleep Quality and Falls Risk in the ELSA Study: A Cross‐Sectional and Prospective Cohort Study
Source: Brain Behav. 2026 Aug 26;16(8):e71719. doi: 10.1002/brb3.71719 (PMC13510640; doi:10.1002/brb3.71719)
Supplement: Supplementary file 5 [file BRB3-16-e71719-s008.pdf]

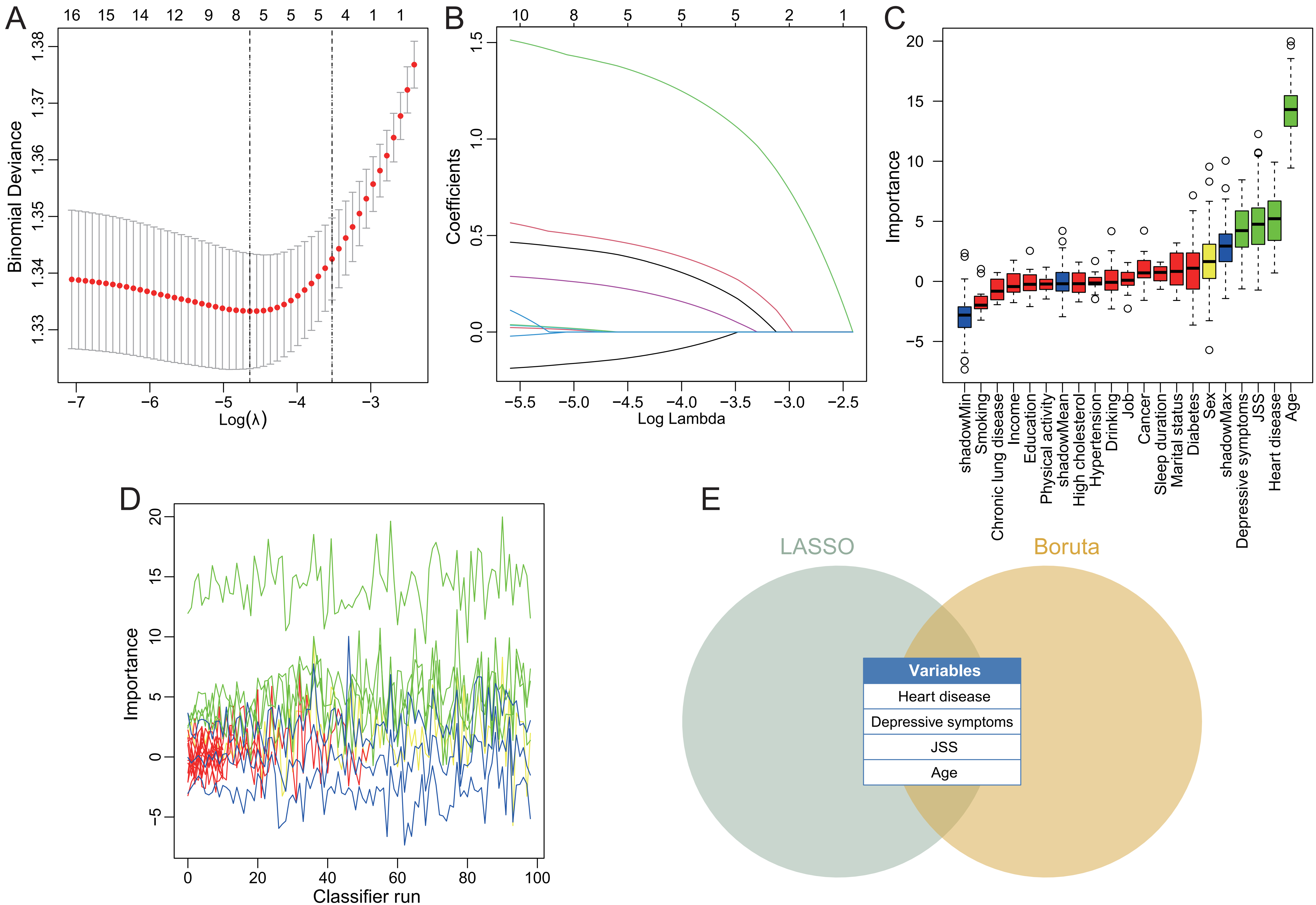


**FIGURE S2** Key variable selection for new-onset fall risk in patients. (A) LASSO regression model factor selection: left dashed line represents the optimal lambda value (lambda.min), while the right dashed line marks the lambda value within one standard error of the optimal (lambda.1se). (B) LASSO regression model screening variable trajectories. (C) Evaluation of variable importance based on the Boruta algorithm. Green for important variables, yellow for tentative ones and red for unimportant ones. (D) The value evolution of importance in the screening process. (E)The Venn diagram of features selected by LASSO and Boruta.
