# Supplementary material for: Sleep Quality and Falls Risk in the ELSA Study: A Cross‐Sectional and Prospective Cohort Study
Source: Brain Behav. 2026 Aug 26;16(8):e71719. doi: 10.1002/brb3.71719 (PMC13510640; doi:10.1002/brb3.71719)
Supplement: Supplementary file 6 [file BRB3-16-e71719-s010.pdf]

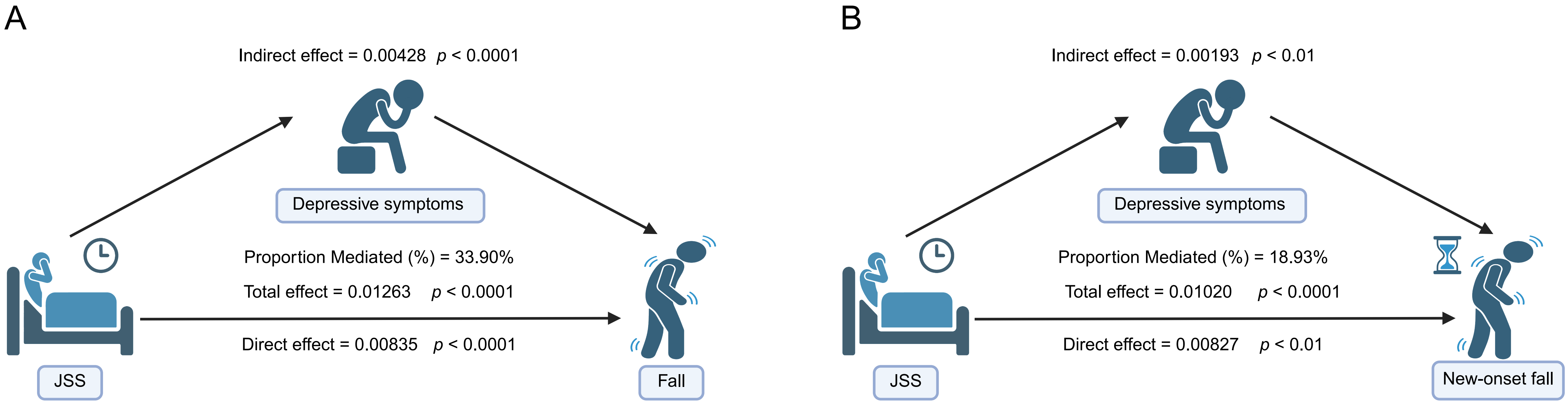

FIGURE S11 Mediation analyses of depressive symptoms in the association between JSS, fall (A) and new-onset fall (B) after excluding participants using hypnotics/sedatives.
