# Supplementary material for: Sleep Quality and Falls Risk in the ELSA Study: A Cross‐Sectional and Prospective Cohort Study
Source: Brain Behav. 2026 Aug 26;16(8):e71719. doi: 10.1002/brb3.71719 (PMC13510640; doi:10.1002/brb3.71719)
Supplement: Supplementary file 8 [file BRB3-16-e71719-s016.pdf]

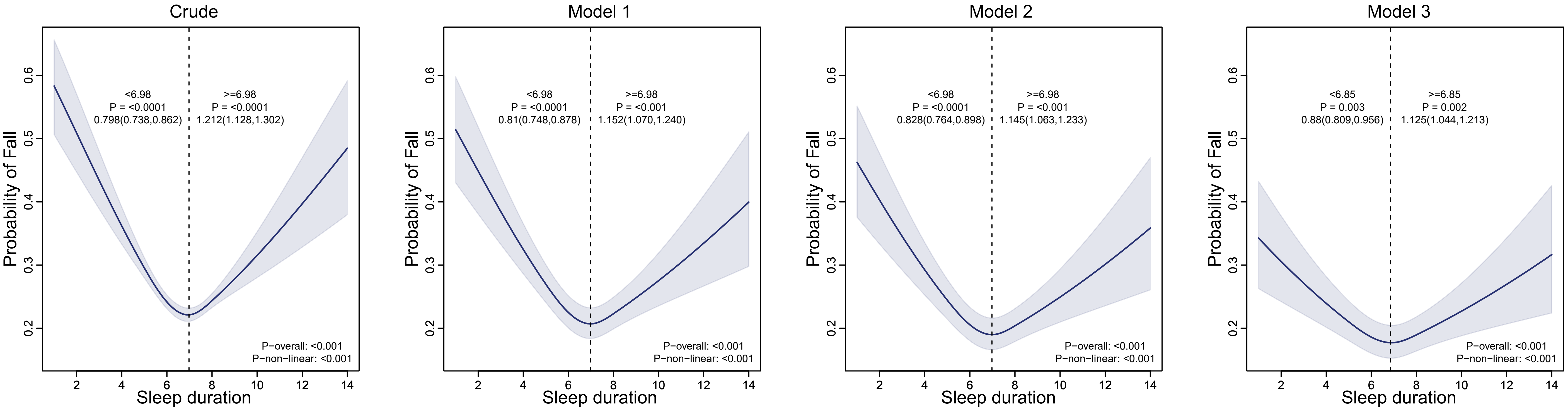

FIGURE S5 Association between sleep duration and fall based on RCS analysis across different models. The x-axis represents the sleep duration, and the y-axis represents the probability.
