# Supplementary material for: Sleep Quality and Falls Risk in the ELSA Study: A Cross‐Sectional and Prospective Cohort Study
Source: Brain Behav. 2026 Aug 26;16(8):e71719. doi: 10.1002/brb3.71719 (PMC13510640; doi:10.1002/brb3.71719)
Supplement: Supplementary file 11 [file BRB3-16-e71719-s022.pdf]

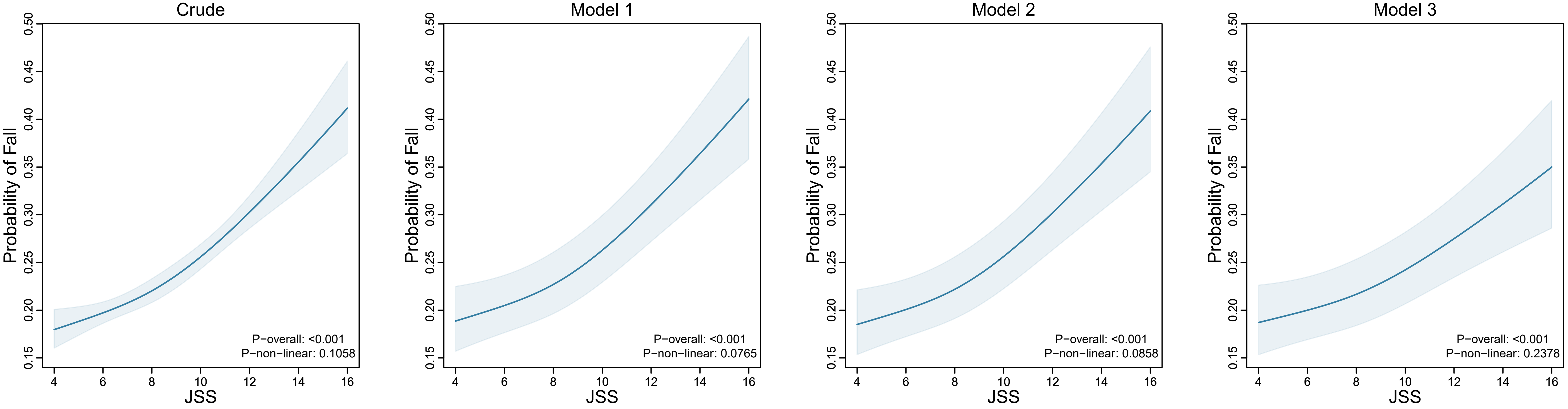

FIGURE S3 Association between JSS and fall based on RCS analysis across different models with normal sleep duration (6-9 hours). The x-axis represents the JSS, and the y-axis represents the probability.
