# Supplementary material for: Sleep Quality and Falls Risk in the ELSA Study: A Cross‐Sectional and Prospective Cohort Study
Source: Brain Behav. 2026 Aug 26;16(8):e71719. doi: 10.1002/brb3.71719 (PMC13510640; doi:10.1002/brb3.71719)
Supplement: Supplementary file 13 [file BRB3-16-e71719-s004.docx]

**Table S3** The mediating effects of the relationship between JSS, and new-onset fall.

|  | **Indirect effect** | **Direct effect** | **Total effect** | **Mediated**  **proportion (%)** | ***p-*value** |
| --- | --- | --- | --- | --- | --- |
|  | **β(95%CI)** | **β(95%CI)** | **β(95%CI)** |  |  |
| Heart disease | 6.18e-04(1.28e-04,0.00) | 9.12e-03(2.69e-03, 0.02) | 9.73e-03(3.26e-03, 0.02) | 6.35 | <0.05 |
| Depressive symptoms | 3.58e-03(1.80e-03,0.01) | 9.11e-03(2.09e-03, 0.02) | 1.27e-02(5.90e-03, 0.02) | 28.20 | <0.01 |
