# Supplementary material for: Sleep Quality and Falls Risk in the ELSA Study: A Cross‐Sectional and Prospective Cohort Study
Source: Brain Behav. 2026 Aug 26;16(8):e71719. doi: 10.1002/brb3.71719 (PMC13510640; doi:10.1002/brb3.71719)
Supplement: Supplementary file 15 [file BRB3-16-e71719-s009.docx]

**Table S7** Multivariate logistic regression analysis of sleep duration and new-onset fall.

| **Variable** | **Crude model** | | **Model 1** | | **Model 2** | | **Model 3** | |
| --- | --- | --- | --- | --- | --- | --- | --- | --- |
|  | **OR(95%CI)** | ***p-*value** | **OR(95%CI)** | ***p-*value** | **OR(95%CI)** | ***p-*value** | **OR(95%CI)** | ***p-*value** |
| Hours | 0.97(0.92,1.03) | 0.35 | 0.99(0.94,1.05) | 0.77 | 0.99(0.94,1.05) | 0.79 | 1.01(0.96,1.08) | 0.62 |
| HoursQ |  |  |  |  |  |  |  |  |
| Q1 | 1(Ref) |  | 1(Ref) |  | 1(Ref) |  | 1(Ref) |  |
| Q2 | 0.87(0.73,1.03) | 0.11 | 0.95(0.79,1.14) | 0.59 | 0.96(0.80,1.15) | 0.68 | 1.02(0.85,1.22) | 0.84 |
| Q3 | 0.94(0.78,1.12) | 0.47 | 1.01(0.84,1.21) | 0.94 | 1.01(0.84,1.22) | 0.89 | 1.08(0.90,1.31) | 0.40 |
| Q4 | 0.96(0.72,1.27) | 0.76 | 0.99(0.74,1.33) | 0.95 | 0.99(0.73,1.32) | 0.92 | 1.03(0.76,1.38) | 0.87 |
| Trend test |  | 0.59 |  | 0.94 |  | 0.93 |  | 0.50 |

OR, odds ratio; CI, confidence interval; Ref: reference

Crude model: Unadjusted

Model 1: Adjusted for age, sex, job, income, education, marital status.

Model 2: Adjusted for age, sex, job, income, education, marital status, physical activity, smoking, drinking.

Model 3: Adjusted for age, sex, job, income, education, marital status, physical activity, smoking, drinking, hypertension, diabetes, high cholesterol, heart disease, chronic lung disease, cancer, depressive symptoms.
