# Supplementary material for: Sleep Quality and Falls Risk in the ELSA Study: A Cross‐Sectional and Prospective Cohort Study
Source: Brain Behav. 2026 Aug 26;16(8):e71719. doi: 10.1002/brb3.71719 (PMC13510640; doi:10.1002/brb3.71719)
Supplement: Supplementary file 16 [file BRB3-16-e71719-s011.docx]

**Table S1** Baseline characteristics of included and excluded populations.

| **Variables** | **Total (n=8009)** | **Excluded (n=4785)** | **Included (n=3224)** | ***p-*value** |
| --- | --- | --- | --- | --- |
| **Age** | 64.31 ± 9.31 | 61.61 ± 9.90 | 68.32 ± 6.56 | <0.0001 |
| **Sex** |  |  |  | 0.21 |
| female | 4327(54.03) | 2613(54.61) | 1714(53.16) |  |
| male | 3682(45.97) | 2172(45.39) | 1510(46.84) |  |
| **Job** |  |  |  | <0.0001 |
| managerial and professional occupations | 1370(17.11) | 1095(22.88) | 275( 8.53) |  |
| intermediate occupations | 940(11.74) | 688(14.38) | 252( 7.82) |  |
| routine and manual occupations | 1133(14.15) | 833(17.41) | 300( 9.31) |  |
| not in work | 4566(57.01) | 2169(45.33) | 2397(74.35) |  |
| **Income** |  |  |  | <0.0001 |
| Q1 | 1591(19.87) | 1056(22.07) | 535(16.59) |  |
| Q2 | 1613(20.14) | 888(18.56) | 725(22.49) |  |
| Q3 | 1601(19.99) | 889(18.58) | 712(22.08) |  |
| Q4 | 1602(20.00) | 917(19.16) | 685(21.25) |  |
| Q5 | 1602(20.00) | 1035(21.63) | 567(17.59) |  |
| **JSS** | 8.68 ± 3.18 | 8.78 ± 3.28 | 8.53 ± 3.01 | <0.001 |
| **Sleep quality** |  |  |  | <0.0001 |
| Poor | 1587(19.82) | 1040(21.73) | 547(16.97) |  |
| Intermediate | 3274(40.88) | 1862(38.91) | 1412(43.80) |  |
| Good | 3148(39.31) | 1883(39.35) | 1265(39.24) |  |
| **Sleep duration** | 6.86 ± 1.29 | 6.83 ± 1.32 | 6.91 ± 1.25 | <0.01 |
| **Education** |  |  |  | <0.01 |
| Below high school | 2491(31.10) | 1429(29.86) | 1062(32.94) |  |
| High school | 2215(27.66) | 1384(28.92) | 831(25.78) |  |
| College or above | 2563(32.00) | 1537(32.12) | 1026(31.82) |  |
| Other | 740( 9.24) | 435( 9.09) | 305( 9.46) |  |
| **Marital** |  |  |  | <0.01 |
| Married or partnered | 5893(73.58) | 3579(74.80) | 2314(71.77) |  |
| Other marital status | 2116(26.42) | 1206(25.20) | 910(28.23) |  |
| **Physical activity** |  |  |  | <0.0001 |
| No | 2799(34.95) | 1773(37.05) | 1026(31.82) |  |
| Yes | 5210(65.05) | 3012(62.95) | 2198(68.18) |  |
| **Smoking** |  |  |  | <0.0001 |
| No | 6826(85.23) | 3936(82.26) | 2890(89.64) |  |
| Yes | 1183(14.77) | 849(17.74) | 334(10.36) |  |
| **Drinking** |  |  |  | <0.0001 |
| No | 1449(18.09) | 875(18.29) | 574(17.80) |  |
| Yes | 4738(59.16) | 2711(56.66) | 2027(62.87) |  |
| Don’t know | 1822(22.75) | 1199(25.06) | 623(19.32) |  |
| **Hypertension** |  |  |  | <0.0001 |
| No | 4000(49.94) | 2572(53.75) | 1428(44.29) |  |
| Yes | 4009(50.06) | 2213(46.25) | 1796(55.71) |  |
| **Diabetes** |  |  |  | 0.01 |
| No | 7156(89.35) | 4309(90.05) | 2847(88.31) |  |
| Yes | 853(10.65) | 476( 9.95) | 377(11.69) |  |
| **High cholesterol** |  |  |  | <0.0001 |
| No | 5643(70.46) | 3485(72.83) | 2158(66.94) |  |
| Yes | 2366(29.54) | 1300(27.17) | 1066(33.06) |  |
| **Heart disease** |  |  |  | 0.02 |
| No | 6622(82.68) | 3997(83.53) | 2625(81.42) |  |
| Yes | 1387(17.32) | 788(16.47) | 599(18.58) |  |
| **Chronic lung disease** |  |  |  | 0.22 |
| No | 7619(95.13) | 4540(94.88) | 3079(95.50) |  |
| Yes | 390( 4.87) | 245( 5.12) | 145( 4.50) |  |
| **Cancer** |  |  |  | 0.04 |
| No | 7409(92.51) | 4451(93.02) | 2958(91.75) |  |
| Yes | 600( 7.49) | 334( 6.98) | 266( 8.25) |  |
| **Depressive symptoms** | 0.96 ± 1.62 | 1.05 ± 1.71 | 0.81 ± 1.48 | <0.0001 |

All values were presented as Mean±SD, or counts (proportion)
