# Supplementary material for: Sleep Quality and Falls Risk in the ELSA Study: A Cross‐Sectional and Prospective Cohort Study
Source: Brain Behav. 2026 Aug 26;16(8):e71719. doi: 10.1002/brb3.71719 (PMC13510640; doi:10.1002/brb3.71719)
Supplement: Supplementary file 17 [file BRB3-16-e71719-s013.docx]

**Table S10** Multivariable logistic regression analysis of sleep quality and fall after excluding participants with hypnotic/sedative use.

| **Variable** | **Crude model** | | **Model 1** | | **Model 2** | | **Model 3** | |
| --- | --- | --- | --- | --- | --- | --- | --- | --- |
|  | **OR(95%CI)** | ***p-*value** | **OR(95%CI)** | ***p-*value** | **OR(95%CI)** | ***p-*value** | **OR(95%CI)** | ***p-*value** |
| JSS | 1.07(1.05,1.09) | <0.0001 | 1.07(1.05,1.10) | <0.0001 | 1.07(1.05,1.09) | <0.0001 | 1.04(1.02,1.07) | <0.001 |
| Sleep quality |  |  |  |  |  |  |  |  |
| Poor | ref |  | ref |  | ref |  | ref |  |
| Intermediate | 0.73(0.62,0.84) | <0.0001 | 0.74(0.63,0.87) | <0.001 | 0.75(0.64,0.89) | <0.001 | 0.86(0.73,1.02) | 0.08 |
| Good | 0.61(0.52,0.71) | <0.0001 | 0.63(0.52,0.75) | <0.0001 | 0.65(0.54,0.77) | <0.0001 | 0.79(0.65,0.95) | 0.01 |
| Trend test |  | <0.0001 |  | <0.0001 |  | <0.0001 |  | 0.02 |

OR, odds ratio; CI, confidence interval; Ref: reference

Crude model: Unadjusted

Model 1: Adjusted for age, sex, job, income, sleep duration, education, marital status.

Model 2: Adjusted for age, sex, job, income, sleep duration, education, marital status, physical activity, smoking, drinking.

Model 3: Adjusted for age, sex, job, income, sleep duration, education, marital status, physical activity, smoking, drinking, hypertension, diabetes, high cholesterol, heart disease, chronic lung disease, cancer, depressive symptoms.
