# Supplementary material for: Sleep Quality and Falls Risk in the ELSA Study: A Cross‐Sectional and Prospective Cohort Study
Source: Brain Behav. 2026 Aug 26;16(8):e71719. doi: 10.1002/brb3.71719 (PMC13510640; doi:10.1002/brb3.71719)
Supplement: Supplementary file 19 [file BRB3-16-e71719-s015.docx]

**Table S2** The mediating effects of the relationship between JSS, and fall.

|  | **Indirect effects** | **Direct effects** | **Total effects** | **Mediated**  **proportion (%)** | ***p-*value** |
| --- | --- | --- | --- | --- | --- |
|  | **β(95%CI)** | **β(95%CI)** | **β(95%CI)** |  |  |
| Heart disease | 4.77e-04(2.50e-04, 0.00) | 1.13e-02(8.23e-03, 0.01) | 1.18e-02(8.70e-03, 0.01) | 4.03 | <0.0001 |
| Depressive symptoms | 4.46e-03(3.41e-03,0.01) | 1.13e-02(8.03e-03, 0.01) | 1.58e-02(1.26e-02, 0.02) | 28.24 | <0.0001 |
