# Supplementary material for: Sleep Quality and Falls Risk in the ELSA Study: A Cross‐Sectional and Prospective Cohort Study
Source: Brain Behav. 2026 Aug 26;16(8):e71719. doi: 10.1002/brb3.71719 (PMC13510640; doi:10.1002/brb3.71719)
Supplement: Supplementary file 20 [file BRB3-16-e71719-s017.docx]

**Table S4** Multivariate logistic regression analysis of sleep quality and fall with normal sleep duration (6–9 hours).

| **Variable** | **Crude model** | | **Model 1** | | **Model 2** | | **Model 3** | |
| --- | --- | --- | --- | --- | --- | --- | --- | --- |
|  | **OR(95%CI)** | ***p-*value** | **OR(95%CI)** | ***p-*value** | **OR(95%CI)** | ***p-*value** | **OR(95%CI)** | ***p-*value** |
| JSS | 1.10(1.08,1.12) | <0.0001 | 1.10(1.08,1.13) | <0.0001 | 1.10(1.08,1.12) | <0.0001 | 1.08(1.06,1.10) | <0.0001 |
| Sleep quality |  |  |  |  |  |  |  |  |
| Poor | 1(Ref) |  | 1(Ref) |  | 1(Ref) |  | 1(Ref) |  |
| Intermediate | 0.66(0.57,0.76) | <0.0001 | 0.64(0.55,0.75) | <0.0001 | 0.65(0.56,0.76) | <0.0001 | 0.72(0.62,0.84) | <0.0001 |
| Good | 0.50(0.43,0.59) | <0.0001 | 0.49(0.42,0.58) | <0.0001 | 0.50(0.43,0.59) | <0.0001 | 0.60(0.50,0.71) | <0.0001 |
| Trend test |  | <0.0001 |  | <0.0001 |  | <0.0001 |  | <0.0001 |

OR, odds ratio; CI, confidence interval; Ref: reference

Crude model: Unadjusted

Model 1: Adjusted for age, sex, job, income, sleep duration, education, marital status.

Model 2: Adjusted for age, sex, job, income, sleep duration, education, marital status, physical activity, smoking, drinking.

Model 3: Adjusted for age, sex, job, income, sleep duration, education, marital status, physical activity, smoking, drinking, hypertension, diabetes, high cholesterol, heart disease, chronic lung disease, cancer, depressive symptoms.
