# Supplementary material for: Sleep Quality and Falls Risk in the ELSA Study: A Cross‐Sectional and Prospective Cohort Study
Source: Brain Behav. 2026 Aug 26;16(8):e71719. doi: 10.1002/brb3.71719 (PMC13510640; doi:10.1002/brb3.71719)
Supplement: Supplementary file 22 [file BRB3-16-e71719-s021.docx]

**Table S6** Multivariate logistic regression analysis of sleep duration and fall.

| **Variable** | **Crude model** | | **Model 1** | | **Model 2** | | **Model 3** | |
| --- | --- | --- | --- | --- | --- | --- | --- | --- |
|  | **OR(95%CI)** | ***p-*value** | **OR(95%CI)** | ***p-*value** | **OR(95%CI)** | ***p-*value** | **OR(95%CI)** | ***p-*value** |
| Hours | 0.93(0.90,0.96) | <0.0001 | 0.93(0.90,0.97) | <0.0001 | 0.94(0.91,0.97) | <0.001 | 0.97(0.94,1.01) | 0.11 |
| HoursQ |  |  |  |  |  |  |  |  |
| Q1 | 1(Ref) |  | 1(Ref) |  | 1(Ref) |  | 1(Ref) |  |
| Q2 | 0.73(0.65,0.81) | <0.0001 | 0.76(0.68,0.86) | <0.0001 | 0.78(0.69,0.88) | <0.0001 | 0.85(0.76,0.96) | 0.01 |
| Q3 | 0.77(0.68,0.87) | <0.0001 | 0.80(0.71,0.90) | <0.001 | 0.82(0.73,0.92) | 0.001 | 0.91(0.81,1.03) | 0.15 |
| Q4 | 1.10(0.93,1.31) | 0.27 | 1.03(0.86,1.22) | 0.75 | 1.03(0.87,1.23) | 0.72 | 1.10(0.92,1.31) | 0.29 |
| Trend test |  | 0.07 |  | 0.06 |  | 0.11 |  | 0.97 |

OR, odds ratio; CI, confidence interval; Ref: reference

Crude model: Unadjusted

Model 1: Adjusted for age, sex, job, income, education, marital status.

Model 2: Adjusted for age, sex, job, income, education, marital status, physical activity, smoking, drinking.

Model 3: Adjusted for age, sex, job, income, education, marital status, physical activity, smoking, drinking, hypertension, diabetes, high cholesterol, heart disease, chronic lung disease, cancer, depressive symptoms.
